# Genito-Urinary and Syphilitic Diseases

**Published:** 1897-09

**Authors:** Byron H. Daggett

**Affiliations:** Buffalo, N. Y.


### GENITO-URINARY AND SYPHILITIC DISEASES.

CONDUCTED BY BYRON H. DAGGETT, M. D., Buffalo, N. Y.

### TREATMENT OF CHRONIC NEPHRITIS.

FAVILL says the etiology of renal degeneration (*Chicago Medical Recorder*, February, 1897,) must be taken into consideration when prescribing treatment. Clinically considered, nephritis is a progressive, nutritive perversion causing necrosis, followed by more or less substitution of one histological form for another. The inflammatory process is present in a small proportion of the cases. Pathologists and clinicians are apparently at variance regarding the question of inflammatory process in chronic nephritis. There are three classes of causes—namely, vascular, septic and toxic.

The principles of therapeusis rest upon these various etiological factors. Organic change is undoubtedly preceded by functional defect, some disturbance of cell capacity.

Can a diagnosis be made previous to anatomical degeneration ? It is possible to make a diagnosis as tissue destruction of the kidney begins and this is the period for therapeutic possibilities.

The treatment of the early stages of nephritis is distinctly different in character from that required for the later stages of this malady, hence the importance of distinguishing the incipient and advanced stages. The primary indication is to relieve the excretory labor of the kidney by prescribing the most digestible food, in order that waste products shall be as small in quantity as possible, and all measures, medicinal, hygienic and others, shall contribute to this end. The most important measure is dietetic. Promote bodily combustion, promote oxidation, a principle or rule of treatment that is simple, but complex in its practical details.

All observers agree that the essential offending element results from the defective oxidised nitrogenous food. The nitrogenous food is the most difficult to oxidise, and with impaired digestion and oxidising power there is a residuum of sub-oxidised products remaining to be eliminated by the enfeebled kidneys.

As the treatment of nephritis is largely dietary, the albuminous proteids should be prohibited, excepting the milk diet; at least the proteid food should be allowed sparingly, governed by the functional, metabolic capacity of the system.

The urea is the basis upon which to estimate the oxidising power, taking into consideration the food, its kind and quantity, as well as the size of the patient.

There are two valuable drugs which promote oxidisation—namely, iron and mercury. In anemic conditions, iron is especially indicated, as it adds to the oxidising machinery of the body. Mercury in the form of calomel is to be administered for months at a time, in from one-twentieth to one-half a grain dose three times daily. It is alterative and metabolic. These two remedies will cover the most of the therapeutical indications.

The treatment of the later stages of nephritis is the treatment of its complications.

---

### GENERAL MANAGEMENT OF CHRONIC NEPHRITIS.

Frank Overton (*The Medical Compend*) states that with the subsidence of the cause of acute nephritis the kidney returns to its normal condition. Chronic nephritis tends to perpetuate itself.

When the kidneys are unable to do their work, some of the energy of the other organs of the body is directed to this purpose, thus impairing digestion, assimilation, elimination and malnutrition results. Inert waste products are not fully oxidised (urea), resulting in the excessive production of uric acid, creatinin, xanthine and other irritants. This calls for more work from the already overburdened kidneys and establishes a vicious circle. Without the primary fault of the kidneys, disturbance of digestion and assimilation may bring about a chronic nephritis. This is exemplified by the moderate and constant user of alcoholic liquors. The constant use of alcohol impairs the oxidation of the albumin of the blood. Suboxidation results in the formation of irritating products which develop nephritis or aggravate preëxisting nephritis.

### PROGNOSIS OF CHRONIC NEPHRITIS.

WALLS (*Chicago Medical Reporter*, February, 1897,) reports that clinicians agree that where the anatomical, pathological lesion of chronic Bright's disease is established, anatomical cure is impossible; yet under certain favoring circumstances the existence of chronic nephritis is not incompatible with a long life of apparent good health. The kidneys are capable of doing many times the work normally exacted of them, and if the lesion comes on slowly a large portion of the kidney structure may be destroyed before the other eliminating organs are called upon to vicariously excrete the urinary products.

Bright's disease is classified under three heads: parenchymatous, interstitial and mixed. The prognosis is nearly always bad in the parenchymatous variety, which terminates fatally in from several months to three years. It is alleged that a relative or functional cure occurs occasionally, when secondary contraction is established, this condition being known as the mixed form.

Death occurs from uremia, dropsy or secondary inflammation of the vital organs in the parenchymatous variety, thus conforming with Osler's paradox that patients seldom die of the disease from which they suffer.

The anatomical lesion of chronic interstitial nephritis is incurable, yet its victim often lives many years in fair health. Dickenson reports cases that lived ten to twenty years.

Uremia, cardiac insufficiency, dropsy, incessant vomiting, diarrhea and respiratory toxemia are storm signals of grave import. Chronic Bright's disease, after scarlet fever and diphtheria, is very fatal, but recovery is possible. The evolution of chronic Bright's disease in the aged is slow, but is none the less grave. Bright's disease in pregnancy during the last month is more favorable if the dangers of acute uremia are circumvented, but a fatal exacerbation may occur after delivery. The condition of the heart, whether compensatory hypertrophy occurs, or muscular impairment is present, prolongs or hastens death. In interstitial nephritis the degree of cardiac hypertrophy indicates the amount of contraction of the kidney. Cardiac impairment indicates a grave prognosis. Cardiac atony is manifested by diminished arterial tension and increased venous tension. Arterial rigidity indicates sclerosis, which is frequently present in the cerebral vessels and predisposes to aneurism, rupture and apoplexy. Impairment of the functions of the skin and alimentary canal, together with acne, gangrene, erysipelas and the like, complicate the prognosis. Albuminuric retinitis, according to Eichhorst, indicates death within two years. Dropsy, due to cutaneous vaso-motor irritation, is less grave in its prognosis than mixed anasarca, due to cardiac insufficiency and blood cachexia. General dropsy, pericardial and pleural effusions render the prognosis very grave. Uremia is a complex symptom, and if it comes on suddenly is a graver prognostic sign than if its onset be slow and gradual. Respiratory phenomena are critical in their significance. Coma, convulsions and delirium indicate an early fatal termination. Albumin, casts, amount of urinary solids, especially urea, are valuable prophetic criteria. They indicate the capacity of the kidneys. Numerous granular, fatty and epithelial casts indicate the degree of kidney destruction. The prognosis of chronic Bright's disease is modified by the concomitant disturbance of other organs of the body.

---

### THE DIAGNOSIS OF CHRONIC NEPHRITIS.

Edwards states that (*Chicago Medical Recorder*, February, 1897,) systematic and repeated examinations of the urine are required in the diagnosis of chronic nephritis. The examination must be made from a twenty-four hour specimen, taking the specific gravity, the gross amount of solids, albumin and microscopic morphology, casts, pus, and the like. Exceptional clinical features sometimes obscure the diagnosis. In the terminal stage of interstitial nephritis, the amount of urine decreases and is constantly small in the absence of cardiac hypertrophy. Persistent low specific gravity and total amount of solids rather indicate functional inadequacy than renal disease. Albumin is found after repeated examination of daily quantity passed.

It is a mistake to regard albuminuria as a certain and constant symptom. Nephritis certainly exists without albuminuria. The diagnosis is made from microscopic and visceral conditions. The presence of uremia, retinal and cardio-vascular lesions are diagnostic symptoms. Indubitable nephritis sine albuminuria has been reported. Casts may be found at intervals when albumin is temporarily absent. Granular and epithelial casts point to some degenerative inflammatory lesion. It is questionable whether even hyaline casts are found in normal urine. Cardio-vascular changes are not invariably present in contracted kidneys. He who examines the heart and urine rarely fails to diagnosticate nephritis.

The heart lesion may be primary or secondary to renal disease. Digitalis and strychnia have a real diagnostic value in differentiation between renal stasis and cardiac activity. Gallop-rhythm characterises renal disease. It is difficult or impossible to differentiate between myocarditis with renal stasis, and renal disease with ultimate cardiac asystole. Differentiation of the various forms of renal disease, parenchymatous and interstitial nephritis, primary and secondary contraction, chronic hemorrhagic nephritis or acute exacerbation of chronic nephritis, contraction or arterio-sclerotic atrophy, is often possible, but impracticable, owing to disagreement of pathologists and clinicians regarding these divisions ; some pathologists only recognise chronic nephritis.

The status and therapeusis differ with the etiology, *e. g.*, nephritis is scarcely the same disease when caused by malaria, pregnancy, tuberculosis, endocarditis or plumbism. The manifold symptoms of other seemingly dominant diseases may obscure ; imperfect history, inefficient physical examination, neglect of known facts, and the like, may lead to a mistaken diagnosis.

Urine albumin may be a sub-oxidised form of serum albumin. Cases of kidney disease, secondary to indigestion and assimilation, are especially dangerous and rapid in their course because unrecognised and because patients are so loath to give up their vicious methods of eating and drinking.

The management of the diet is of primary importance. The first indication is to diminish the amount of nitrogenous products to be excreted. This is done by diminishing the amount of nitrogenous food, and promoting oxidation by means suggested elsewhere. The giving of amylaceous and fatty foods without albumin does not remedy the difficulty, since oxidation of these products will occur at the expense of the tissues of the body. A definite amount of albumin is oxidised each day, so that if it is not supplied by the food, it is taken from the system. It is impossible to control the nitrogenous excretory products by prohibiting the use of albuminous food. If the amount of sugar and fat be limited, and an abundant diet of easily digested albuminoids be eaten, the food will be easily oxidised, thus lessening the amount of irritating waste products to be eliminated. Milk is the typical food. Later something more substantial is required, then add sugar, fat, eggs, bread and butter and fresh meat.

Assist digestion. To aid the stomach give dilute hydrochloric acid. To aid intestinal digestion give chologogues, combined with a mercurial, ox bile, strychnia, extract of colocynth compound and pancreatin. Give sufficient colocynth to insure two to three movements of the bowels each day.

A select diet fortified by digestive aids, with a good quantity of food, will be assimilated with the least expenditure of energy and with the least amount of irritating waste products. Aid excretion by mercury and colocynth acting upon the liver and intestines. Give diuretics, strychnia, digitalis and caffeine, and avoid the depressing and irritating urinary diuretics, such as potassium salts, buchu, turpentine, and the like.

In acute exacerbations give croton oil, elaterium and stimulate the skin with hot air bath and pilocarpin. For anasarca and dyspnea give compound jalap powder. Administer full doses of these remedies to insure prompt results.

Even after albumin and casts have disappeared, and the urine is normal in quantity, but low in specific gravity (urinary insufficiency), continue the dieting, so as to forestall recrudescence of the disease. In this way this malady will be held in check for years.

Dr. Elliott says that the mixed diet is the most rational. Meat should constitute one-fourth and vegetables three-fourths of the total amount. Over-indulgence gives to the circulation a large amount of nitrogenous waste, to be eliminated by the kidneys, overtaxed by a hyperacid (concentrated urine), since these subjects seldom drink an adequate amount of water. The by-products of indigestion from over-indulgence add to the irritation and eventually lead to organic impairment of the kidneys.

---

### METHYLENE BLUE IN THE DIAGNOSIS OF RENAL PERMEABILITY.

M. ACHARD stated in a report published in the *Journal des Practiciens* that he had gathered fifty new observations and performed eighteen autopsies, which confirm results previously obtained by him. He verified the integrity of the kidneys by autopsies in twenty-two cases of normal elimination of methylene blue. Thirteen out of twenty-eight cases of tardy elimination showed renal lesions at the autopsy.

In a case of nephrydrosis it was shown by ureteral catheterisation, that methylene blue did not pass through the diseased kidney. Ordinarily there were no therapeutical effects due to the methylene blue on the albuminuria.

M. Hirtz states that he has employed this remedy in six cases of albuminuria and observed no appreciable benefit.

M. Chamtemesse gave twelve to fifteen grains a day without results.

Netchaieff reports a case of Bright's disease in a man aged fifty years, which presented the cardiac and renal difficulties as well as the albuminuria and casts characterising this malady. He alleges that methylene blue given three times daily in one and one-half grain doses relieved all the symptoms. Albumin and casts disappeared.—*Therapeutic Gazette.*

